# Supplementary figures and images for: COMMIT: Consideration of metabolite leakage and community composition improves microbial community reconstructions
Source: PLoS Comput Biol. 2022 Mar 23;18(3):e1009906. doi: 10.1371/journal.pcbi.1009906 (PMC8942231; doi:10.1371/journal.pcbi.1009906)

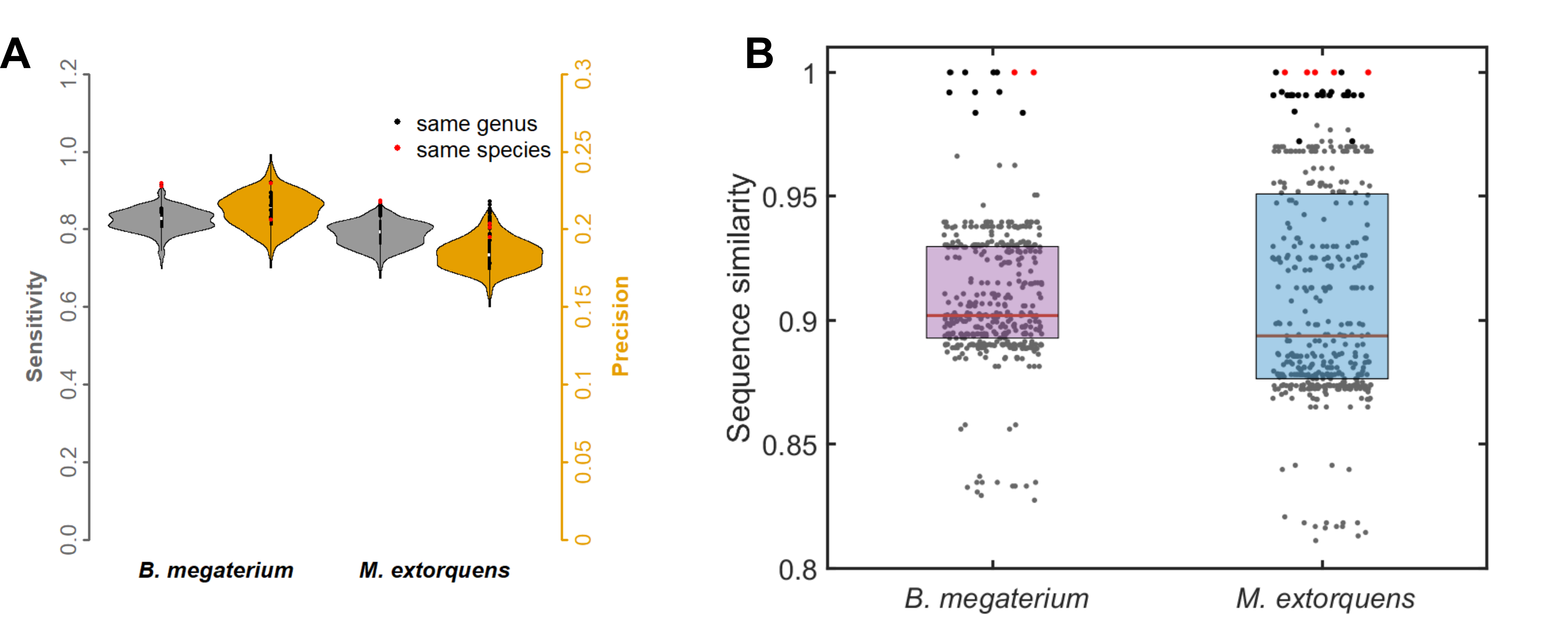

Supplement: S1 Fig — (A) The sensitivity (left) and precision (right) with respect to metabolite and E.C. number sets were calculated for each of the 432 reconstructions based on the two reference models. (B) Sequence similarity of 16S rRNA sequences of isolates to the ones of the two reference species. The red line depicts the median and the box limits represent the 25% and 75% quartiles, respectively. The black dots indicate isolates that were assigned the same genus (9 for Bacillus megaterium and 27 for Methylobacterium extorquens) according to Bai et al. [38]. Isolates that were predicted to belong to the same species are shown as red dots (2 for B. Megaterium and 5 for M. extorquens). (PNG) [file pcbi.1009906.s001.png]

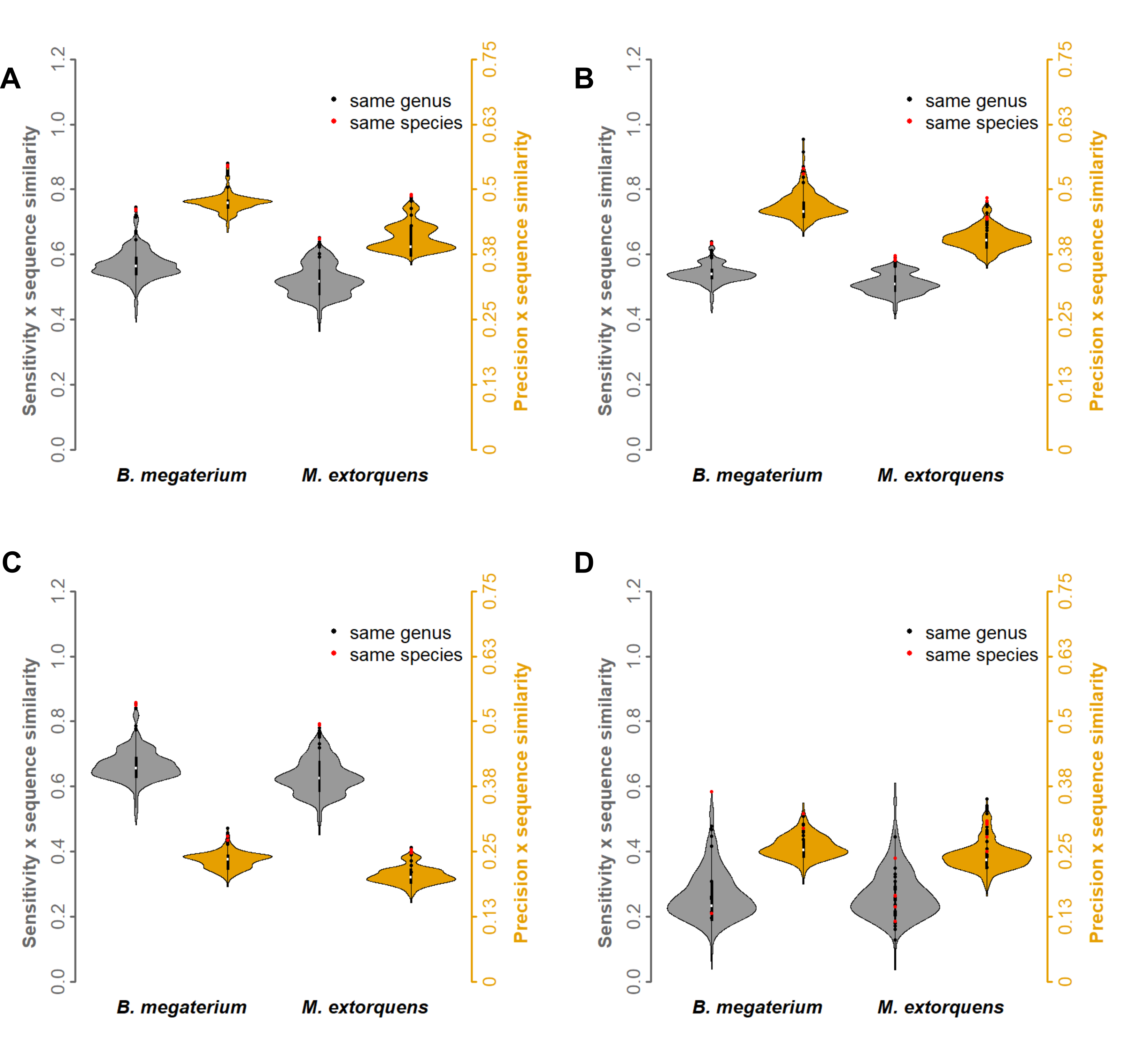

Supplement: S2 Fig — The sensitivity (left) and precision (right) with respect to metabolite and E.C. number sets were calculated for each of the 432 reconstructions based on the two reference models. These values were scaled by the sequence similarity to the 16S rRNA sequences of the used references. (A) KBase [13] (B) CarveMe [14] (C) RAVEN 2.0 [17], and (D) AuReMe/Pathway Tools [15,16]. The black dots indicate isolates that were assigned the same genus (9 for Bacillus megaterium and 27 for Methylobacterium extorquens) according to Bai et al. [38]. Isolates that were predicted to belong to the same species are shown as red dots (2 for B. Megaterium and 5 for M. extorquens). (PNG) [file pcbi.1009906.s002.png]

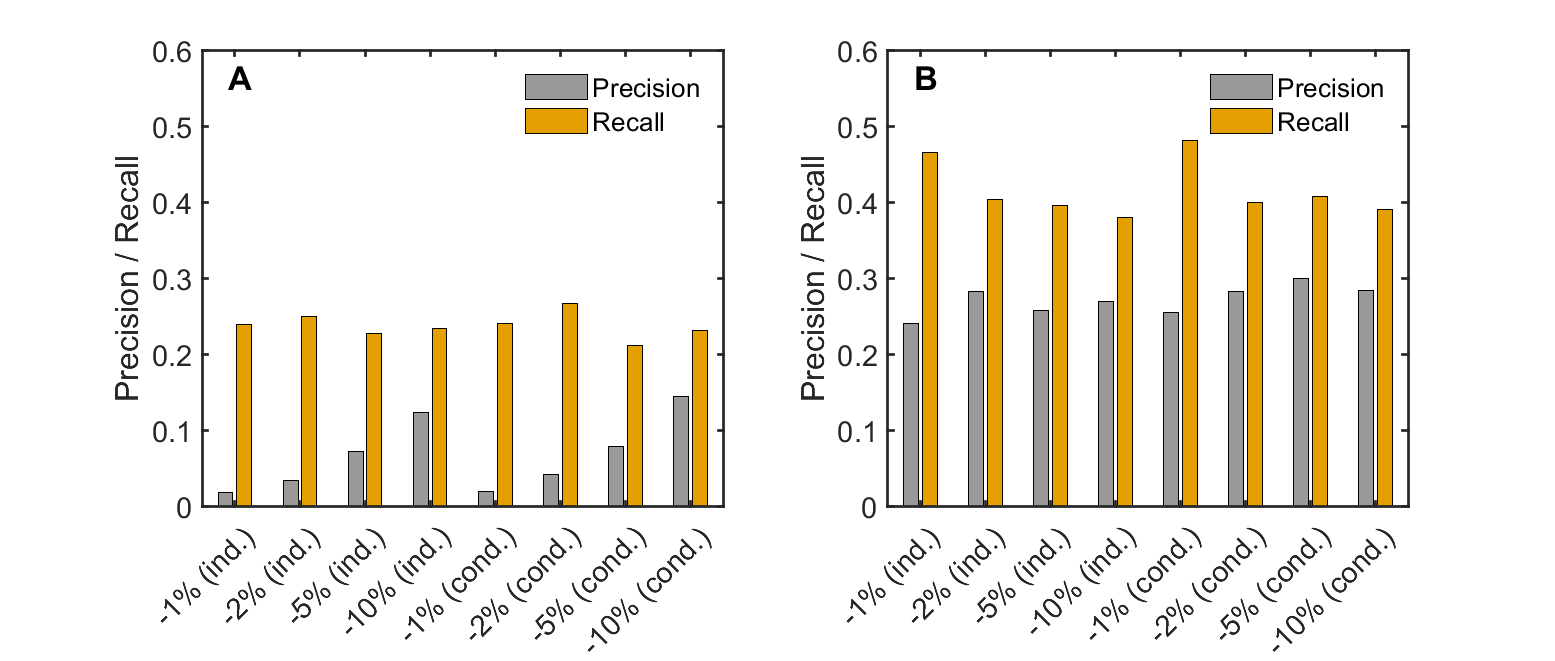

Supplement: S3 Fig — COMMIT was run with (cond.) and without (ind.) consideration of the community composition, i.e. taking permeable metabolites into account. The procedure of removing random reactions (1, 2, 5, and 10 percent) was repeated 50 times for (A) the Desulfovibrio vulgaris [56] and (B) the Methanococcus maripaludis [55] metabolic models. We only allowed flux-carrying, internal reactions to be removed, which could also be translated to the MNXref namespace (341 for D. vulgaris and 304 for M. maripaludis). (PNG) [file pcbi.1009906.s003.png]

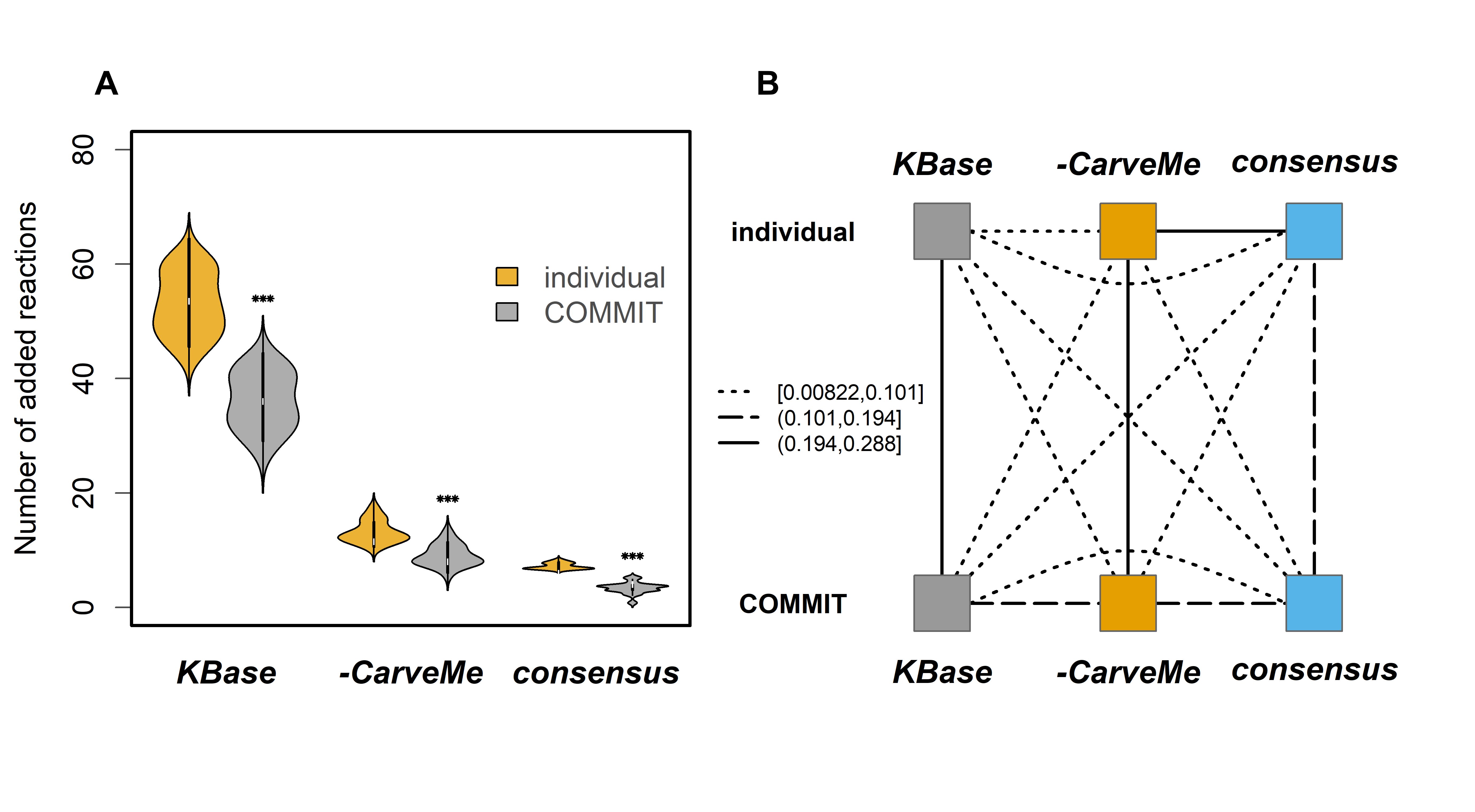

Supplement: S4 Fig — Full consensus (consensus), consensus without CarveMe reconstructions (-CarveMe), and KBase draft reconstructions (KBase) were gap-filled either individually or using the COMMIT approach. (A) Sizes of gap-filling solution sets were compared for each reconstruction type using a paired Wilcoxon rank sum test (*** p<0.001). (B) Pairwise comparison of added reactions obtained for each reconstruction and gap-filling type by calculating the Jaccard similarity per isolate. The resulting matrices were merged per group using the STATIS method [40–42]. The obtained values were grouped using K-means clustering (K = 3). The line types indicate the average similarity between the compared groups. (PNG) [file pcbi.1009906.s004.png]

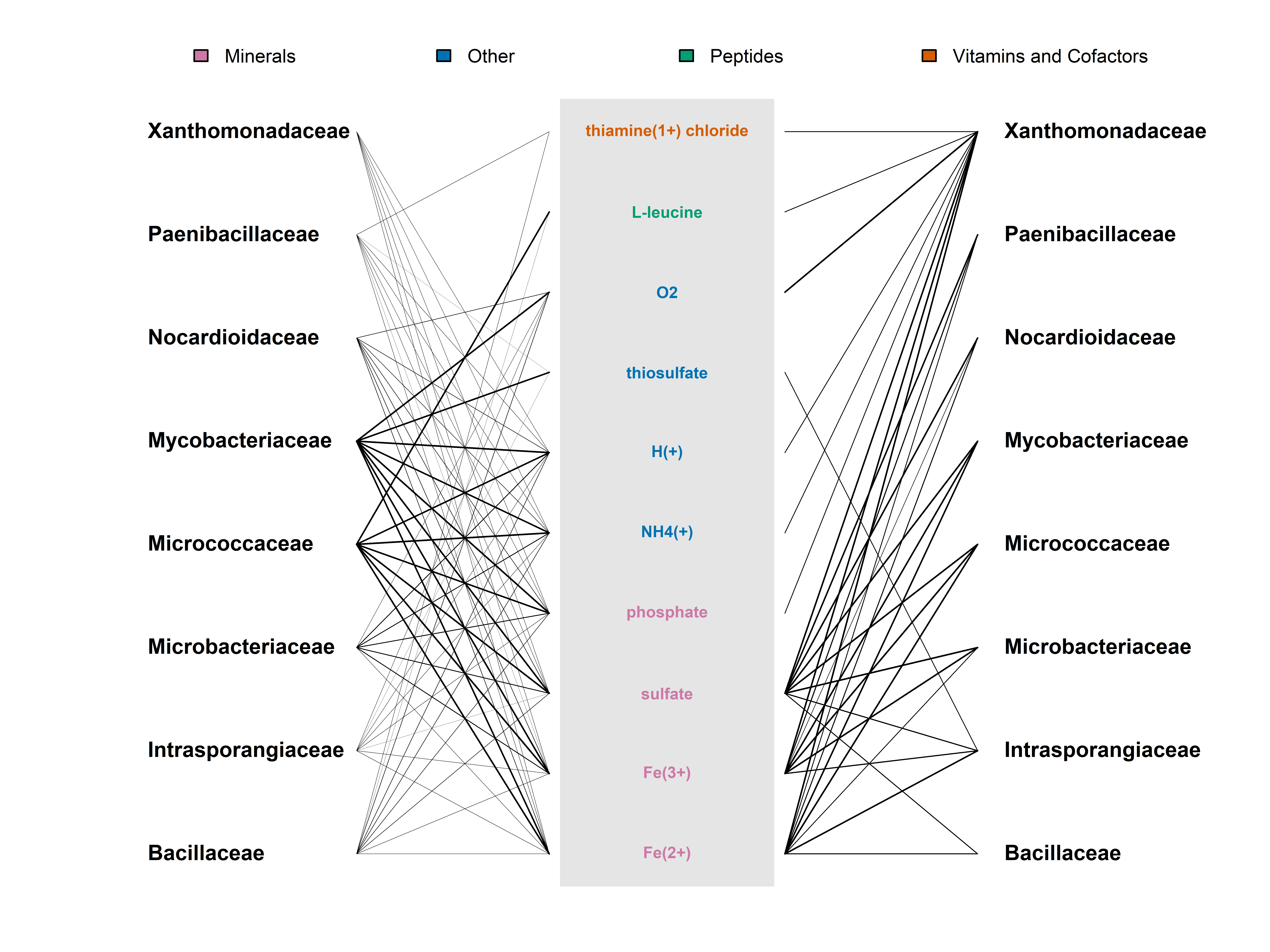

Supplement: S5 Fig — The sets of imported and secreted metabolites that were determined for each member during conditional gap-filling and grouped into corresponding bacterial families. On the left-hand side, metabolite export is shown, which does not require a reduction of growth greater than 10%. The common pool of exchanged metabolites is shown in the center, which were classified using the KEGG BRITE br08001 with manual refinement. On the right-hand side, import reactions are shown, which were introduced during the gap-filling procedure. Line widths represent the number of community members per import/export of a metabolite, scaled by the abundance of the respective family. (PNG) [file pcbi.1009906.s005.png]

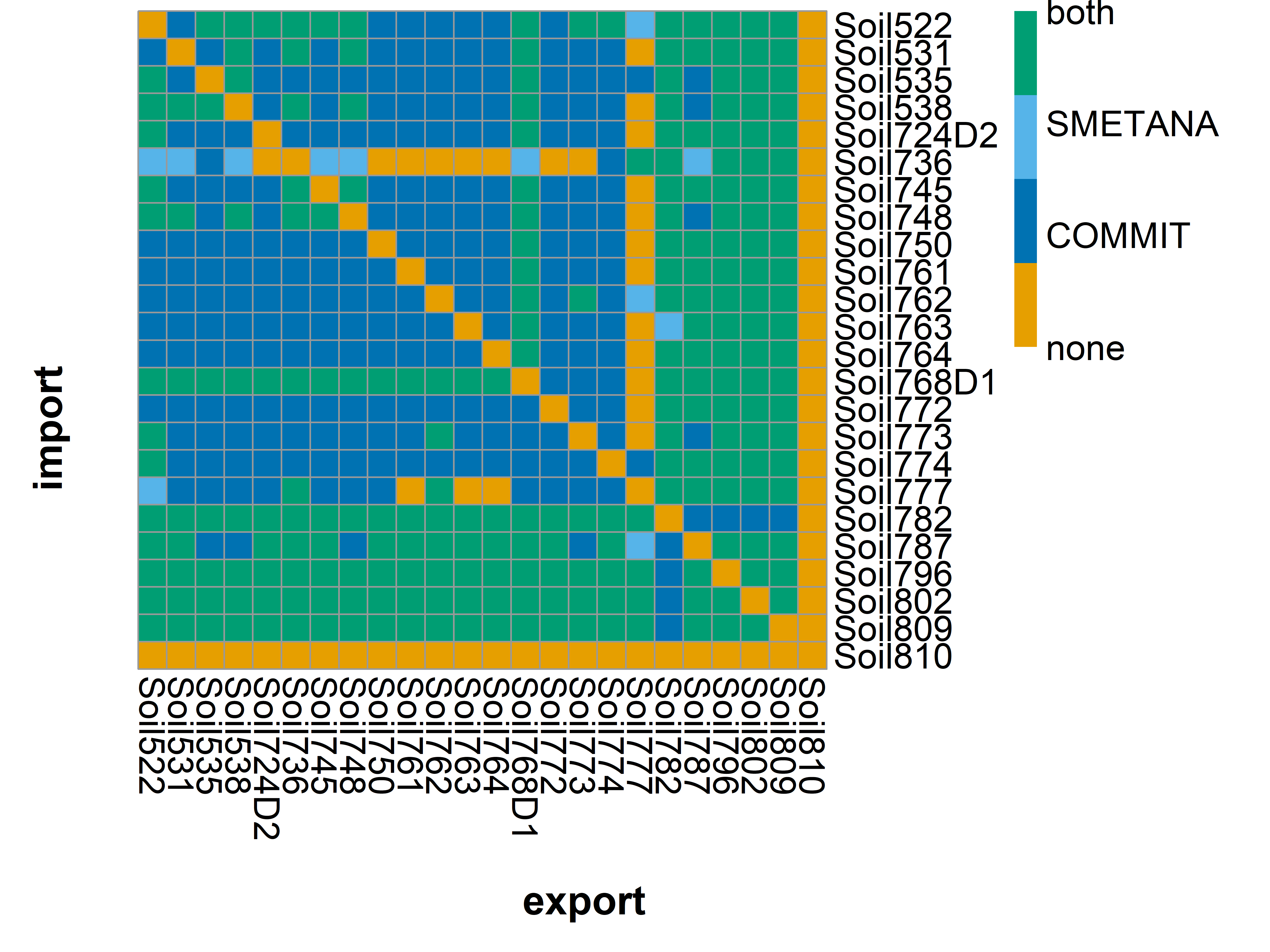

Supplement: S6 Fig — An interaction between two community members was defined as non-empty overlap between the respective sets of imported and exported metabolites. As a result, we obtained a directed graph, in which we scored whether an edge was present with both (green), either one of the methods (light or dark blue) of none of the methods (orange). The directed exchanges returned by SMETANA [10] (implementation from github.com/cdanielmachado/smetana) were transformed to undirected interactions by taking the pairwise overlap of imported and exported metabolites for each pair of reconstructions. (PNG) [file pcbi.1009906.s006.png]

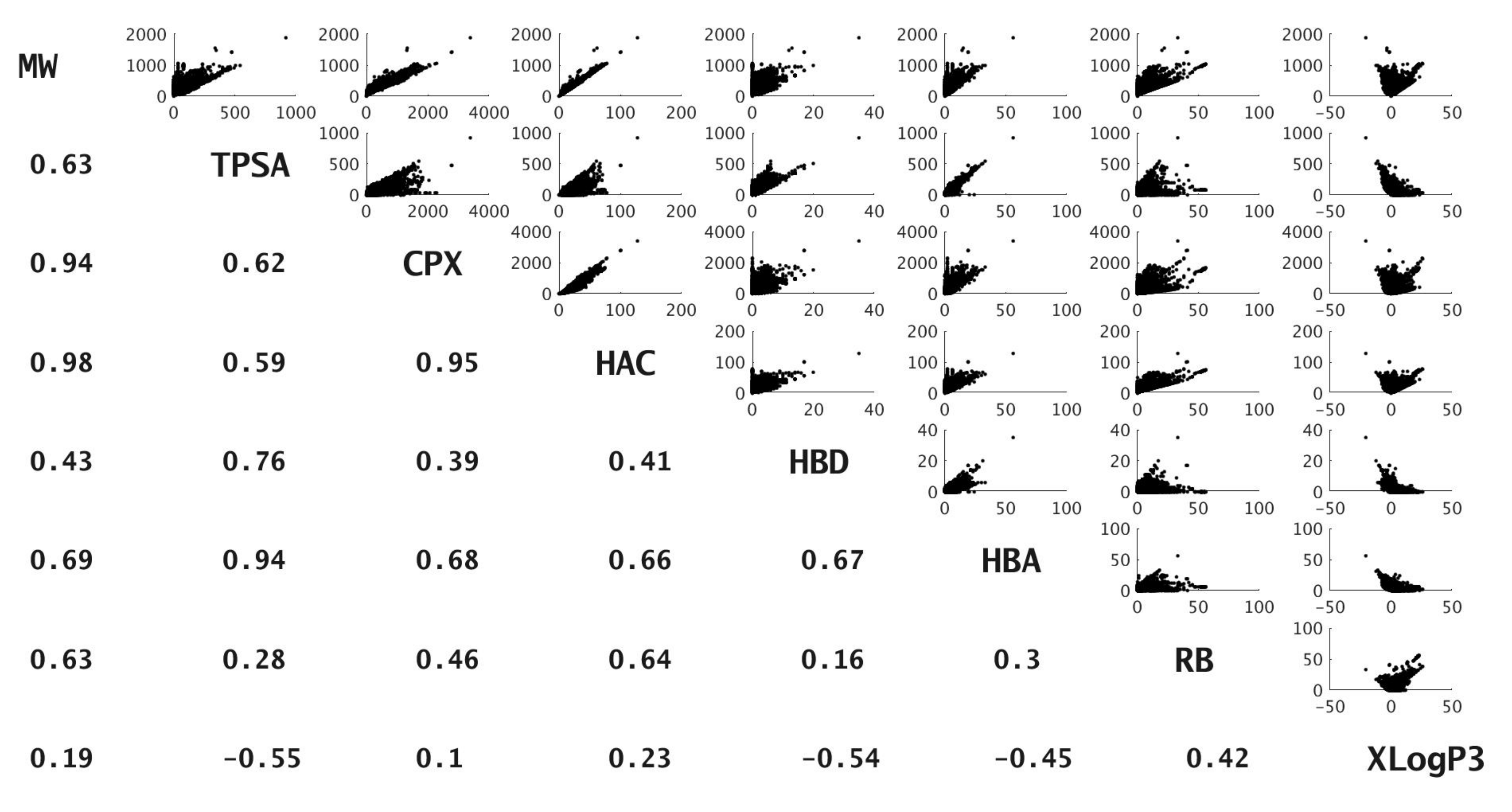

Supplement: S7 Fig — The correlation between molecular weight (MW), polar surface area (TPSA), complexity (CPX), heavy atom count (HAC), numbers of H-bond donors (HBD), H-bond acceptors (HBA) and rotatable bonds, and the predicted XlogP3 values are shown as scatter plots in the upper right triangle and the pearson correlation is given in the lower left triangle. (PNG) [file pcbi.1009906.s007.png]

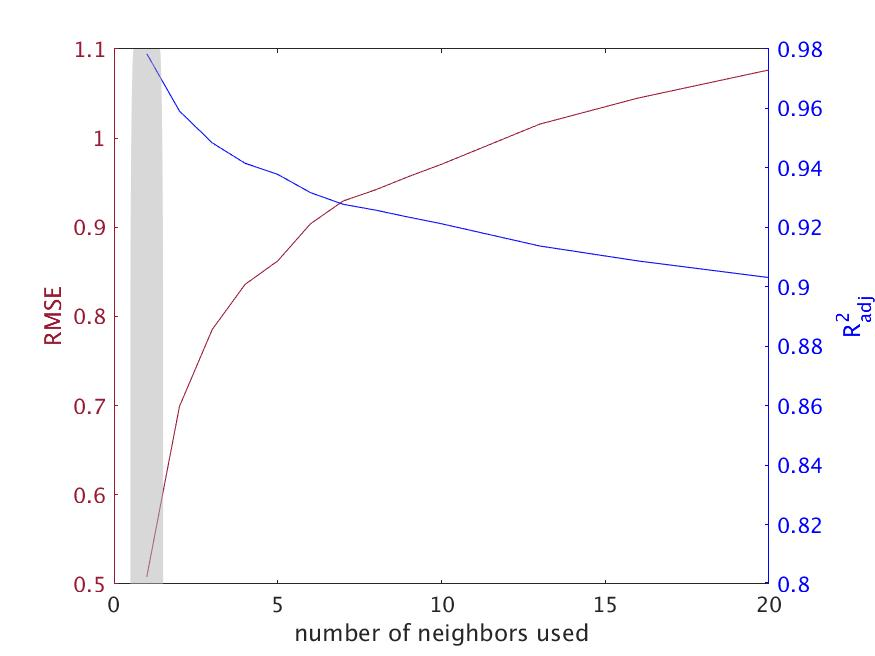

Supplement: S9 Fig — The root mean square error (RMSE) and adjusted R-squared value were compared between kNN regressions with different values for k. The utilized regression model with k = 1 is highlighted in light grey. (PNG) [file pcbi.1009906.s009.png]
