# Supplementary material for: COMMIT: Consideration of metabolite leakage and community composition improves microbial community reconstructions
Source: PLoS Comput Biol. 2022 Mar 23;18(3):e1009906. doi: 10.1371/journal.pcbi.1009906 (PMC8942231; doi:10.1371/journal.pcbi.1009906)
Supplement: S1 Text — Detailed information on the generation of draft reconstructions, distance measures for model comparison, consensus generation, and the pseudocode of the COMMIT procedure. Table A in S1 Text. Abundances and numbers of exchanged metabolites of all models in the Schlaeppi community. The abundances were summed up between the environmental samples investigated in the study after normalization [36]. The numbers of imported and exported metabolites are given as their set difference to the medium. Further, only highly-permeable metabolites were considered, respectively. Table B in S1 Text. Abundances and numbers of exchanged metabolites of all models in the Bulgarelli community. The abundances were summed up between the environmental samples investigated in the study after normalization [37]. The numbers of imported and exported metabolites are given as their set difference to the medium. Further, only highly-permeable metabolites were considered. Table C in S1 Text. Minimal medium used for gap filling. Medium, which has been used for conditional and individual gap filling of the metabolic reconstructions used in this study. It is composed of nutrients that were predicted as required by all models used in both communities. Table D in S1 Text. Quality assessment using the MEMOTE test suite [75]. Unfortunately, we were not able to obtain memote scores for RAVEN 2.0 reconstructions. Table E in S1 Text. ChEBI metabolite ontology enrichment of the highly-permeable metabolites in the gap-filling database. An enrichment analysis of ChEBI metabolite ontology terms was used to compare metabolites, which have been predicted to be highly permeable were compared to all metabolites in the gap-filling database. The p-values have been corrected for multiple testing using the Benjamini-Hochberg procedure. The terms are further sorted by their occurrence in the highly-permeable metabolite set. (PDF) [file pcbi.1009906.s010.pdf]

S1 Text

Generation of draft reconstructions

Four different reconstruction approaches were used for genome-scale metabolic reconstruction generation: KBase/ModelSEED [1,2], RAVEN 2.0 [3], AuReMe/Pathway Tools v19 [4,5] and CarveMe [6]. Within the KBase web application, the genome assemblies were RAST-annotated [7] using the 'annotate_contigset' function using code cells. Further, draft reconstructions and auxotrophy predictions were generated using 'build_multiple_metabolic_models' and 'predict_genome_auxotrophy'.

The RAVEN 2.0 reconstruction pipeline was applied with the amino-acid FASTA files for all OTUs. The required Hidden Markov models (HMM) were generated based on KEGG Orthology (KO) files for prokaryotic organisms obtained from KEGG v90 [8] with a 90% protein redundancy cut-off. For multiple sequence alignments (MSA), MAFFT v7.427 software [9] was used. Within MATLAB [10], default settings were used for the RAVEN 2.0 functions for automated reconstruction of KEGG and MetaCyc reconstructions. As an exception, the number related species considered for sequence similarity search was set to 30. Moreover, spontaneous, stoichiometrically-undefined and incomplete reactions were excluded from the draft reconstructions.

AuReMe draft reconstructions were generated using its annotation-based reconstruction method (Docker image *aureme-img:2.1*). To this end, the genome assemblies were first annotated using DFAST [11] and then Pathway Tools data files were obtained for all annotations. These were converted to *PADMet* files to reconstruct the draft reconstructions with AuReMe. Finally, as a fourth approach, CarveMe was used with default settings with the amino-acid FASTA files as inputs. Further processing and evaluation of the obtained draft reconstructions was done with MATLAB using in-house functions as well as functions provided by the COBRA toolbox [12] as described below.

Distance measures applied to compare draft reconstructions

SVD distance The singular values of the stoichiometric matrices $S(i)$ for each reconstruction i are first determined and scaled to the respective maximum. The distance between two reconstructions is then assessed by the statistic of the two-sample Kolmogorov-Smirnov test, which quantifies the distance between the respective distributions of scaled singular values [13].

Reaction Jaccard distance (JD) For a pair of reconstructions i and j, the Jaccard distance of reaction sets is calculated by:

$$d_{AB} = 1 - \frac{A \cap B}{A \cup B} \tag{1}$$

with A and B being the reaction sets of reconstructions i and j, respectively.

Metabolite JD As in Eq. 1

$$d_{AB} = 1 - \frac{A \cap B}{A \cup B} \quad (1)$$

the Jaccard distance is calculated for the metabolite sets of the reconstructions i and j.

Dead-end metabolite JD The Jaccard distance between the sets of dead-end metabolites for reconstructions i and j are calculated as in Eq. 1.

E.C. number JD The set for E.C. numbers for each two reconstructions are compared as in Eq. 1 for each pair of reconstructions i and j.

Dead-end metabolite number distance The set of all dead-end metabolites, denoted by DE, is obtained for each reconstruction by the COBRA function 'detectDeadEnds' [12]. The number of dead-end metabolites, $|DE|$ is then scaled to the number of metabolites, m, in the respective reconstruction. The distance between reconstructions i and j is then calculated as:

$$d_{ij}^{nDE} = abs\left(\frac{|DE_i|}{m_i} - \frac{|DE_j|}{m_j}\right). \quad (2)$$

E.C. number occurrence distance For this distance measure, the sets of E.C. numbers for two reconstructions i and j are compared to the set of all EC numbers retrieved from BRENDA [14] and corrected with KEGG for possible updates. The occurrence of each E.C. number is calculated and scaled by the ratio between the number of reactions and the number of E.C. numbers:

$$EC^{tot'}_{i} = \left(1 - \frac{|R_i|}{|EC^{tot}|}\right) \cdot EC_i^{tot}. \quad (3)$$

The resulting vectors EC_i^{tot} and EC_j^{tot} are compared using the Spearman rank correlation coefficient:

$$d_{ij}^{nEC} = 1 - corr\left(EC^{tot'}_{i}, EC^{tot'}_{j}\right), \quad (4)$$

yielding the E.C. number occurrence distance.

Cofactor usage distance A list of cofactors (KEGG br08001: 'Vitamins and Cofactors': 'Cofactors': 'Coenzymes') was obtained from KEGG and translated to MNXref name space [15] for the different reconstruction approaches. First, the occurrence of every compound that classifies as a cofactor is calculated and scaled by the number of reactions in the respective reconstruction. The distance is then obtained in a similar way as in Eq. 4 by calculating the Spearman's rank correlation of the resulting vectors.

Correlation of distance measures to sequence dissimilarity

The distance matrices D obtained using the above-described methods were compared to the respective sequence dissimilarity distance matrix S_j extracted from the Newick tree for each habitat j. The similarity of each pair D_i and S_j was calculated using the Mantel coefficient [16].

$$r_M = \frac{trace\left(D_i' S_j'^{T}\right)}{\sqrt{trace\left(D_i' D_i'^{T}\right) \cdot trace\left(S_j' S_j'^{T}\right)}}, \quad (5)$$

with $D_i' = D_i - d_{iE}$ and $S_j' = S_j - s_{jE}$. The variables d_i and s_i are the means of the off-diagonal elements in D_i and S_j, respectively, and E is a matrix of all ones.

Consensus generation

The merging of reconstructions was done in a step-wise process by starting with the first reconstruction as the initial consensus reconstruction and subsequently comparing the following reconstructions to it: First, the union of all fields is built. Gene identifiers are then compared to the consensus, adding previously unseen genes if necessary. Metabolites are unified in the third step, based on identity of identifiers as the MetaNetX database already matches metabolites based on their structure. The fourth step comprises pairwise comparison of reactions on the basis of identifiers, metabolite composition, mass-balance, reversibility (with preference of irreversible reactions), directionality (distinguishing forward and backward direction of the same reaction), and similarity of gene rules. Cosine similarity C served as a first measure to determine which reactions to compare ($C \geq 0.9$). Reaction pairs with $0.9 \leq |C| < 1$ were additionally tested for missing metabolites and protons, preferring the usage of metabolites in MNXref namespace and inclusion of mass- and charge-balanced reactions. In the case that two reactions have opposite directions, both are kept in the consensus reconstruction. If two reactions are indistinguishable, the reaction that is already contained in the consensus is kept, while the second is discarded. In case both compared reactions are not mass-balanced, the corresponding mass-balanced database reaction is added. The similarity of gene rules is calculated as the product of Jaccard indices of genes and operators. Duplicated reactions are deleted but gene rules of both reactions were merged if not identical.

Full COMMIT procedure

Pseudocode

Input

Set of k models G

initial gap-filling medium M_{gf}

auxotrophic media for each community member M_{aux}

reference database DB

number of iterations n

Output:

gap-filled metabolic models GF

list of exchanged metabolites EX

optimal gap filling order gf_order

initialize(GF, EX, gf, exc, bio)

for i from 1 to n do:

$S_i \leftarrow$ randomOrdering(k)

$P \leftarrow \emptyset$

$M \leftarrow M_{aux,S_{i1}}$

for j from 1 to k do:

$GF_j =$ condFastGapFilling$\left(G[S_{ij}],\ M,\ DB\ \cup\ EX\right)$

$P =$ findPotentialExcMets$\left(GF_{ij}\right) \setminus M$

$EX\ =\ EX \cup P$

$exc_{ij} = |P|$

$bio_{ij} =$ FBA$\left(GF_j\right)$ // *find optimal biomass flux*

$gf_{ij} =$ ReactionNumber$\left(GF_{ij}\right) -$ ReactionNumber$\left(G_{ij}\right)$

$M \leftarrow M_{gf}$

end

$dep_i = \left|M_{aux,S_{i1}} \cap P\right| \cdot \left|M_{aux,S_{i1}}\right|^{-1}$

end

$gf = \sum_i^n gf_i.$

$bio = \sum_i^n bio_i.$

$exc = \sum_i^n exc_i.$

$gf\_order = \min\limits_{gf,-dep,-bio,-exc} S$

The inner loop is repeated with gf_order

1. Generate n random orderings (if no ordering was given)
2. After each gap filling, extend the set of potential exchanged metabolites that can be added during gap filling
3. The best ordering is determined as follows: Find the best solution by first considering orderings that minimize the number of added reactions. Next, the set of orderings is narrowed down iteratively until only one ‘optimal’ ordering remains. To this end, the following criteria have been considered one after the other: maximum dependency of the first model on the exported metabolites of subsequent models, maximum number of exchanged metabolites, and maximum sum of biomass fluxes. If there exist multiple ordering that are ‘optimal’ with respect to these criteria, one of them is selected at random.
4. Gap-fill the reconstructions in the optimal order with respect to the criteria described above.

Table A. Abundances and numbers of exchanged metabolites of all models in the Schlaeppi community. The abundances were summed up between the environmental samples investigated in the study after normalization [17]. The numbers of imported and exported metabolites are given as their set difference to the medium. Further, only highly-permeable metabolites were considered, respectively.

OTU ID	Abundance	Export	Import
**Soil522**	143	58	1
**Soil531**	1199	79	2
**Soil535**	11	66	4
**Soil538**	79	84	3
**Soil724D2**	13	68	3
**Soil736**	45	68	15
**Soil745**	747	78	2
**Soil748**	16	59	3
**Soil750**	4	60	3
**Soil761**	10	61	2
**Soil762**	1	68	3
**Soil763**	2	64	3
**Soil764**	16	69	2
**Soil768D1**	487	73	5
**Soil772**	40	53	3
**Soil773**	4	49	4
**Soil774**	649	61	3
**Soil777**	41	75	1
**Soil782**	3	64	2
**Soil787**	30	65	3
**Soil796**	69	65	5
**Soil802**	194	60	3
**Soil809**	86	51	3
**Soil810**	824	59	3

Table B. Abundances and numbers of exchanged metabolites of all models in the Bulgarelli community. The abundances were summed up between the environmental samples investigated in the study after normalization [18]. The numbers of imported and exported metabolites are given as their set difference to the medium. Further, only highly-permeable metabolites were considered.

OTU ID	Abundance	Export	Import
**Soil522**	19	62	1
**Soil531**	25	80	2
**Soil535**	6	68	3
**Soil538**	18	84	3
**Soil724D2**	5	68	2
**Soil728**	33	54	2
**Soil736**	8	76	3
**Soil745**	12	79	0
**Soil748**	6	67	4
**Soil750**	10	60	3
**Soil768D1**	26	70	2
**Soil772**	6	46	14
**Soil773**	2	49	4
**Soil774**	245	61	2
**Soil777**	9	67	2
**Soil787**	3	65	3
**Soil796**	36	65	2
**Soil802**	227	59	4
**Soil809**	8	52	2
**Soil810**	80	61	2

Table C. Minimal medium used for gap filling. Medium, which has been used for conditional and individual gap filling of the metabolic reconstructions used in this study. It is composed of nutrients that were predicted as required by all models used in both communities.

**MetaNetX ID**	**Metabolite name**
MNXM2	H2O
MNXM11	diphosphate
MNXM95120	iron
MNXM41	D-glucose
MNXM2255	Mn(2+)
MNXM149	Zn(2+)
MNXM632	Cu(2+)
MNXM128	Ca(2+)
MNXM89	sulfur
MNXM43	chloride
MNXM304	biotin
MNXM531993	Molybdenum
MNXM90960	Co(2+)
MNXM95	K(+)
MNXM3673	Ni(2+)
MNXM653	Mg(2+)
MNXM27	Na(+)
MNXM4505	Cd(2+)
MNXM19009	Pb
MNXM161163	sodium
MNXM160440	potassium
MNXM160414	dipotassium
MNXM161213	sodium
MNXM43229	Ba(2+)
MNXM82680	Sr(2+)
MNXM88705	vanadium(5+)
MNXM90852	Methylcobalamin
MNXM157678	magnesium
MNXM161119	sodium
MNXM152150	aluminium(3+)

MNXM160410	potassium
MNXM153069	calcium

Table D. Quality assessment using the MEMOTE test suite [19]**.** Unfortunately, we were not able to obtain *memote* scores for RAVEN 2.0 reconstructions.

Approach	Model ID	Total score	Consistency score	Unbounded Flux in Default Medium
**AuReMe/Pathway Tools**	Leaf183	18	35	100
	Leaf210	18	34	100
	Leaf245	34	76	100
	Leaf291	18	34	100
	Leaf363	17	32	100
	Leaf399	34	75	100
	Leaf9	17	32	100
	Root553	18	35	100
	Root635	33	73	100
	Root83	18	35	100
**CarveMe**	Leaf154	16	33	30.3
	Leaf8	17	33	23.8
	Root170	15	29	57.9
	Root280D1	16	32	32.3
	Root322	15	30	48
	Root53	16	31	38.4
	Root9	16	31	44.5
	Root901	16	31	41.9
	Soil728	16	31	42.1
	Soil809	16	32	34
**KBase**	Leaf159	36	71	97.5
	Leaf186	36	71	95.5
	Leaf222	36	71	95.9
	Leaf233	35	71	98
	Leaf394	36	73	84.8
	Root241	36	72	92.3
	Root418	36	71	95.2
	Root420	36	72	88
	Root554	36	72	94.5
	Root73	36	72	89.8
**Consensus (Schlaeppi)**	Soill522	34	43	70.2
	Soil531	34	43	73.9
	Soil535	35	44	65.1
	Soil538	34	42	75.9
	Soil724D2	34	43	67.9
	Soil736	35	44	66.1
	Soil745	34	43	70.9
	Soil748	35	44	66.8
	Soil750	34	42	75.1
	Soil761	35	44	65.8
	Soil762	35	44	65.7

Soil763	35	44	66.1
Soil764	35	45	61.9
Soil768D1	34	42	74.2
Soil772	35	44	63.3
Soil773	35	44	61.7
Soil774	35	44	63.1
Soil777	34	43	71
Soil782	35	44	63.2
Soil787	34	43	67.1
Soil796	34	43	70.8
Soil802	35	44	62.7
Soil809	35	44	63.8
Soil810	35	44	63.2

Table E. ChEBI metabolite ontology enrichment of the highly-permeable metabolites in the gap-filling database. An enrichment analysis of ChEBI metabolite ontology terms was used to compare metabolites, which have been predicted to be highly permeable were compared to all metabolites in the gap-filling database. The p-values have been corrected for multiple testing using the Benjamini-Hochberg procedure. The terms are further sorted by their occurrence in the highly-permeable metabolite set.

ChEBI ID	Ontology	adjusted p-value
36587	organic oxo compound	$7.3 \cdot 10^{-4}$
36586	carbonyl compound	$4.2 \cdot 10^{-4}$
25696	organic anion	$4.2 \cdot 10^{-9}$
33273	polyatomic anion	$3.3 \cdot 10^{-6}$
25741	oxide	$3.3 \cdot 10^{-6}$
35406	oxoanion	$3.7 \cdot 10^{-5}$
24913	isoprenoid	$7.2 \cdot 10^{-5}$
17087	ketone	$3.9 \cdot 10^{-5}$
29067	carboxylic acid anion	$3.1 \cdot 10^{-5}$
26873	terpenoid	$1.1 \cdot 10^{-4}$
3992	cyclic ketone	$1.4 \cdot 10^{-4}$
35757	monocarboxylic acid anion	$2.4 \cdot 10^{-4}$
35381	monosaccharide	$1.8 \cdot 10^{-4}$
25901	pentose	$9.2 \cdot 10^{-4}$
63944	macrocyclic lactone	$6.2 \cdot 10^{-5}$
26188	polyketide	$6.2 \cdot 10^{-5}$
36401	cycloalkadiene	$4.0 \cdot 10^{-4}$
136889	5beta steroid	$4.0 \cdot 10^{-4}$
26872	terpene ketone	0
23849	diterpenoid	0
22195	acetyl-amino acid	0
28965	dicarboxylic acid dianion	0
77636	fatty acyl-CoA(4-)	0
132539	fatty acid 20:4	0
37613	cyclohexadiene	0
24973	ketohexose	0
33257	secondary amide	0
33760	hexonate	0

82830	sphingosine-based sphingolipid	0
23449	cyclic peptide	0
19260	2'-deoxyribonucleotide	0
37016	2'-deoxyribonucleoside 5'-phosphate	0
61940	aroyl-CoA	0
61912	branched-chain fatty acyl-CoA	0
37015	ribonucleoside 5'-phosphate	0
24971	ketohexose monophosphate	0
19257	2'-deoxyribonucleoside monophosphate	0
26392	purine nucleoside monophosphate	0
26438	pyrimidine nucleoside monophosphate	0
26558	ribonucleoside monophosphate	0
26441	pyrimidine nucleotide	0
22080	TDP-sugar	0
22290	aldaric acid	0
63437	aldaric acid derivative	0
36520	oligoglycosylceramide	0
36498	galactosylceramide	0
35746	fatty aldehyde	0
66873	C4-dicarboxylic acid	0
48847	heterocyclic fatty acid	0
140345	hydroxy polyunsaturated fatty acid	0
15904	long-chain fatty acid	0
23931	epoxy monocarboxylic acid	0
24727	hydroxynaphthalene	0
17522	alditol	0
23229	chromanol	0
33666	polycyclic hydrocarbon	0
33581	boron group molecular entity	0
27024	toluenes	0
22580	anthraquinone	0
26144	piperazines	0
48901	thiazoles	0
24156	galactosamine	0

SI References

1. Henry CS, DeJongh M, Best AA, Frybarger PM, Linsay B, Stevens RL. High-throughput generation, optimization and analysis of genome-scale metabolic models. Nat Biotechnol. 2010;28: 977–982. doi:10.1038/nbt.1672
2. Arkin AP, Cottingham RW, Henry CS, Harris NL, Stevens RL, Maslov S, et al. KBase: The United States Department of Energy Systems Biology Knowledgebase. Nat Biotechnol. 2018;36: 566–569. doi:10.1038/nbt.4163
3. Wang H, Marcišauskas S, Sánchez BJ, Domenzain I, Hermansson D, Agren R, et al. RAVEN 2.0: A versatile toolbox for metabolic network reconstruction and a case study on *Streptomyces coelicolor*. PLOS Comput Biol. 2018;14: e1006541. doi:10.1371/journal.pcbi.1006541
4. Aite M, Chevallier M, Frioux C, Trottier C, Got J, Cortés MP, et al. Traceability, reproducibility and wiki-exploration for “à-la-carte” reconstructions of genome-scale metabolic models. Nielsen J, editor. PLOS Comput Biol. 2018;14: e1006146. doi:10.1371/journal.pcbi.1006146
5. Karp PD, Latendresse M, Paley SM, Krummenacker M, Ong QD, Billington R, et al. Pathway Tools version 19.0 update: software for pathway/genome informatics and systems biology. Brief Bioinform. 2016;17: 877–890. doi:10.1093/bib/bbv079
6. Machado D, Andrejev S, Tramontano M, Patil KR. Fast automated reconstruction of genome-scale metabolic models for microbial species and communities. Nucleic Acids Res. 2018;46: 7542–7553. doi:10.1093/nar/gky537
7. Brettin T, Davis JJ, Disz T, Edwards RA, Gerdes S, Olsen GJ, et al. RASTtk: a modular and extensible implementation of the RAST algorithm for building custom annotation pipelines and annotating batches of genomes. Sci Rep. 2015;5: 8365. doi:10.1038/srep08365
8. Kanehisa M, Goto S. KEGG: kyoto encyclopedia of genes and genomes. Nucleic Acids Res. 2000;28: 27–30. doi:10.1093/nar/28.1.27
9. Katoh K, Standley DM. MAFFT multiple sequence alignment software version 7: improvements in performance and usability. Mol Biol Evol. 2013;30: 772–780.
10. MATLAB. version 9.3.0 (R2017b). Natick, Massachusetts: The MathWorks Inc.; 2017.
11. Tanizawa Y, Fujisawa T, Nakamura Y. DFAST: a flexible prokaryotic genome annotation pipeline for faster genome publication. Bioinformatics. 2018;34: 1037–1039. doi:10.1093/bioinformatics/btx713
12. Heirendt L, Arreckx S, Pfau T, Mendoza SN, Richelle A, Heinken A, et al. Creation and analysis of biochemical constraint-based models using the COBRA Toolbox v.3.0. Nat Protoc. 2019;14: 639–702. doi:10.1038/s41596-018-0098-2
13. Omranian N, Kleessen S, Tohge T, Klie S, Basler G, Mueller-Roeber B, et al. Differential metabolic and coexpression networks of plant metabolism. Trends Plant Sci. 2015;20: 266–268.
14. Jeske L, Placzek S, Schomburg I, Chang A, Schomburg D. BRENDA in 2019: a European ELEXIR core data resource. Nucleic Acids Res. 2019;47: D542–D549.
15. Moretti S, Martin O, Van Du Tran T, Bridge A, Morgat A, Pagni M. MetaNetX/MNXref - Reconciliation of metabolites and biochemical reactions to bring together genome-scale metabolic networks. Nucleic Acids Res. 2016;44: D523–D526. doi:10.1093/nar/gkv1117
16. Mantel N. The Detection of Disease Clustering and a Generalized Regression Approach. Cancer Res. 1967;27: 209–220. Available: https://cancerres.aacrjournals.org/content/27/2_Part_1/209
17. Schlaeppi K, Dombrowski N, Oter RG, van Themaat E, Schulze-Lefert P. Quantitative divergence of the bacterial root microbiota in *Arabidopsis thaliana* relatives. Proc Natl Acad Sci. 2014;111: 585–592. doi:10.1073/pnas.1321597111
18. Bulgarelli D, Rott M, Schlaeppi K, van Themaat E, Ahmadinejad N, Assenza F, et al. Revealing structure and assembly cues for *Arabidopsis* root-inhabiting bacterial microbiota. Nature. 2012;488: 91–95. doi:10.1038/nature11336
19. Lieven C, Beber ME, Olivier BG, Bergmann FT, Ataman M, Babaei P, et al. MEMOTE for standardized genome-scale metabolic model testing. Nat Biotechnol. 2020;38: 272–276. doi:10.5281/zenodo.2636858
