# Supplementary material for: COMMIT: Consideration of metabolite leakage and community composition improves microbial community reconstructions
Source: PLoS Comput Biol. 2022 Mar 23;18(3):e1009906. doi: 10.1371/journal.pcbi.1009906 (PMC8942231; doi:10.1371/journal.pcbi.1009906)
Supplement: Attachment — Submitted filename: Review_response.pdf [file pcbi.1009906.s011.pdf]

Reviewer #1

Reviewer #1: The authors present a tool that gap-fills individual microbial metabolic reconstructions based on the metabolic reconstructions of organisms found in the same community. The COMMIT algorithm starts with gap-filling a single random metabolic model from the community on minimal media. COMMIT takes into account the permeability of metabolites when deciding on the set of gap-filled reactions. After finding the optimal gap-filling reaction set, it simulates the maximum biomass flux of the model and adds the list of secreted metabolites to the media for gap-filling the next randomly chosen model, and so on, until all models have been gap-filled. COMMIT metabolic reconstructions contain fewer gap-filled reactions than their individually gap-filled counterparts from other sources. COMMIT is a novel gap-filling algorithm that considers both metabolite permeability and other members of the microbial community, which are important factors to consider when modeling microbial metabolism. While I see many uses for this method, I also have a few concerns and questions about the general applicability of the metabolic reconstructions that are produced by the algorithm.

Major points

If you have an organism that is found in multiple different communities, how different is the resulting metabolic reconstruction? With COMMIT, the gap-filling reactions are dependent on the community, so supposedly the end-result would differ. With that in mind, can a COMMIT reconstruction of an organism be used in different applications or only within the context of that particular community?

To the first question, the final reconstructions for the same organism across different communities is expected to change with respect to the composition of the communities, i.e. depending on the nutrients available from the environment and those produced by other community members. Therefore, the final reconstructions obtained from COMMIT can be seen as community-specific reconstructions, akin to the context-specific reconstructions [Robaina Estévez and Nikoloski, 2014 (10.3389/fpls.2014.00491)], of an underlying blueprint reconstruction for a given organism.

Since the community-specific reconstructions can only differ in the reaction set that was added during gap-filling, we determined the Jaccard Index between the reactions added to the reconstructions from the Bulgarelli and Schlaeppi communities. We found the highest Jaccard Index for the KBase reconstructions with a median of 0.64, and lower values for consensus models without CarveMe reconstructions (0.6) and full consensus models (0.5). Here, one has to consider that the number of reactions added to the full consensus models is considerably lower than with the other reconstruction types, which usually only include uptake reactions.

Regarding the second question, we concur that resulting models can be used outside of the community context. This can be done by either only applying the first step of COMMIT that entails merging the reconstructions from the four approaches, followed by gap-filling in another context of choice, or by gap-filling the models with multiple community compositions first and then taking the consensus of added reactions as the complete model for further analysis.

It is not clear how much (if any) of this work is manual. The authors talk about selecting the optimal set of secreted metabolites based on summed growth rate and number of gap-filled reactions, but it is not clear if this is automatically done by the algorithm or if this step is manual.

COMMIT is fully automated as the goal of this study was to find a method that generates sufficiently accurate metabolic models from samples. Thus, all steps from reconstruction and consensus generation to gap-filling and the selection of optimal solution sets is automated within modules. The selection of the final, optimal ordering of models is done by subsequently filtering for those orderings that (1) minimize the number of added reactions, (2) show the strongest dependence of the first model on exchanged metabolites from the rest of the community, (3) maximize the number of exchanged metabolites, and (4) maximize the overall of biomass production of the community.

Some general terminology needs reworking regarding the use of the phrases metabolic model vs. reconstruction. The authors frequently use “model” where “reconstruction” would be more appropriate. In short, a reconstruction is the set of reactions, genes, and metabolites, whereas a model is a reconstruction with applied constraints and is used for simulations.

We thank the reviewer for this helpful comment. We updated the manuscript in the appropriate places accordingly.

The authors associate reconstruction quality by the number of gaps, but I disagree with that assessment. Depending on what you want to learn from the reconstruction or its models, it’s good to have gaps as they tell you where there is a lack of knowledge of the microbe’s metabolism.

We would like to thank the reviewer for the critical statement, and we agree that the number of gaps in a reconstruction *per se* cannot serve as a quality measure. Without additional manual curation a reconstruction will definitely contain many gaps. However, as a part of an automated curation process, the union of multiple reaction databases for an organism can already help close a number of gaps in the network that would have to be otherwise closed by manual curation or gap-filling otherwise. We changed the wording in the updated manuscript, where we now separate the expectation for model quality improvement from the reduction in the number of gaps on the path to biomass production.

L112-114: How come KBase, CarveMe and RAVEN 2.0 were more similar to each other? Do they build on the similar annotations, databases, or algorithms? Looking at the lower triangle in Fig. 1A, how come AuReMe is so completely different from the others? They look about 90% different, what is the reason the gene differences are so big? How come RAVEN is so similar to the consensus but the others seem quite distant?

Regarding the first question, we would like to note that all reconstructions from the four approaches showed relatively high similarity with respect to the eight structural measures that we compared. All

four reconstruction approaches are based on different functional annotation strategies and biochemical databases used (except for RAVEN 2.0 and AuReMe/Pathway Tools, which both include MetaCyc). The reason why AuReMe/Pathway Tools reconstructions are that different from those obtained by the other methods is because the gene and reaction sets of these reconstructions are much smaller compared to the other approaches; as a result, the Jaccard distance is affected by the rather large size difference and hence small intersection of the sets that are compared. This is further due to the observation that AuReMe/Pathway Tools reconstructions only contain reactions with gene association.

The high similarity of RAVEN 2.0 reconstructions to the consensus is likely a result of the size of RAVEN 2.0 draft reconstructions, especially with respect to metabolites and reactions, but also genes (Fig. 1C). Consequently, the proportion of shared features with the consensus is higher. The reason for the increased size of the models is a high number of duplicated reactions, which was assessed by translation of MetaCyc and KEGG reaction identifiers to MNXref (average of 41.5%).

Figure 1B and 1C: I don't understand these figures; they need clearer explanations. What is the orange Sum boxplot showing? It is not clear from the figure or caption.

We thank the reviewer for this remark. The orange box in Figure 1C represents the number of reactions, metabolites, or genes in a hypothetical consensus model without reconciliation, i.e. it corresponds merely to the sum of these features from all four draft reconstructions. We updated the captions in the updated version of the manuscript.

How does a consensus model from just the species-specific reconstructions compare to the genus-specific reconstructions (red dots in Fig. 1D and Fig. S1)? Is a consensus model from all isolates useful?

Figure 1D does not show the performance of genus- or species-specific consensus models. We computed the sensitivity and precision measures between each isolate-specific model and the respective reference model. The red and black dots show whether the respective isolate-specific models share the same genus or species with the reference model. We reworked the caption of Figure 1D and S1 in the updated version.

What is the unscaled sensitivity of the reconstructions in Fig. 1D and S1 and how does the sequence similarity compare among all the genomes? I would expect that the genus-specific genomes are less similar than the species-specific ones, so how does that affect the scaling? Is the sensitivity similar among all and it's the sequence similarity that is dragging the genus-specific reconstructions down in the plots, or a combination of lower sequence similarities and lower sensitivity?

We thank the reviewer for this critical observation. We included a figure that includes the unscaled values to show that the sensitivity and precision those reconstructions for isolates that share only the

genus is not strongly drawn down by scaling with sequence similarity. We noted that the ordering of precision values for the *B. megaterium* model changes by scaling, so the reconstructions that share the same genus or species have higher values relative to the remaining reconstruction compared to the unscaled values. We further added a figure that depicts the sequence similarity of all isolates to the reference sequences. The main reason for including the sequence similarity in this plot was to emphasize the difference between values for reconstructions for the same species, same genus, and the remaining ones.

L336-337: The manuscript states a “strong reduction” here, whereas in Fig. 6 the corresponding field is yellow, which according to the caption indicates “a smaller reduction of biomass”. Also, I would expect that the R. thiooxydans model dependency on sulphur was already present in the draft model, is this not the case? I don’t see how gap-filling the reconstruction (i.e., adding reactions) could introduce a dependency; for that, reactions must be removed from the network or directionality changed from reversible to irreversible (usually only the change from irreversible to reversible is done in gap-filling).

The reviewer is correct in noting that the wording was not appropriate in the first version of the manuscript. However, due to the modifications of the approach in the revised version of the manuscript, this observation no longer holds, so it was removed from the text. Therefore, we do not comment further to this point.

L340-342: I feel that this sentence is overstated in the current version of the manuscript. High-confidence models are compared with experimental data of multiple metabolites, both in terms of uptake and secretion, but this kind of benchmarking has not been done in this study. These models are potentially high confidence, but without some sort of validation for all models for multiple metabolites, I feel that the authors cannot make this statement.

We updated the sentence in the revised manuscript.

Minor points

L130-133: Model quality assessed with number of gaps. Why is Memote not used for assessing model quality here? It’s used later in the manuscript.

When consensus generation is regarded as a way to close knowledge gaps, a reduction of gaps can be regarded as an improvement of quality. We included Memote reports for models from the original draft reconstructs from the four approaches in Table S4.

L145-147: Why does RAVEN have larger networks and AuReMe smaller? Are they higher/lower in gap-filled reactions? Was the periplasm compartment accounted for in the comparisons like it was in the consensus models?

The comparisons were conducted using the converted reconstructions, i.e. the periplasm compartment was already treated as extracellular. RAVEN 2.0 reconstructions are larger than those generated by other methods, because this pipeline already created consensus models from the KEGG and MetaCyc databases. Hence, more reactions are included in the merged RAVEN model, which are non-overlapping between both draft models or cannot be reconciled. We did not fill gaps in single draft reconstructions (except for KBase models), but we do not expect a big difference in the number added reactions under the assumption that all models cover central carbon metabolism rather well and miss out parts of the secondary metabolism.

L160-161: What does “low translation efficiency” refer to? Reaction/metabolite/gene translation into MXNref? Also, what is the reference point for “low” here? 30%? 50%? 70%?

Yes, the translation efficiency refers to reaction and metabolite translation to MNXref. We revisited this part of the manuscript and found additional metabolite identifiers in the supplementary material for the *B. megaterium* model. These could be translated with 99.7% efficiency to MNXref identifiers and about 70% of the reactions in the model have assigned E.C. numbers, which were also used for the comparison. The translation efficiencies for *M. extorquens* metabolite identifiers were 96% (KEGG) and 96% (MetaCyc). Further, 51% of the reactions have E.C. numbers assigned, which are also used in the comparisons.

L161-163: keep in mind that both reference models were built using a draft reconstruction combined from KEGG (used by RAVEN) and Model SEED (uses RAST like KBase), and in the case of B. megaterium, a pre-existing network that I cannot access, and the paper is only available in a foreign language, so I cannot check its origin.

We are aware of the model building process that both reference models have undergone; however, the final model structure did not contain any cross-references for metabolites and reactions. We now included KEGG identifiers for the metabolites that we found in the supplementary material.

L193: The use of exported and exchanged metabolites is confusing. Perhaps rephrasing “exported” metabolites to “secreted” might make the text clearer?

We thank the reviewer for the suggestion, we improved the nomenclature in the updated manuscript.

L198: The new definition of “exchangeable metabolite” is unfortunate here given the traditional phrase for a metabolic reconstruction boundary reaction is “exchange reaction”. Is there any other phrase that could make the distinction between the two clearer?

In the rephrased version of the manuscript, we use the term “permeable metabolites” instead of “exchangeable metabolites”.

L237-239: I find Fig. 3 generally difficult to understand. It is not clear how the scaling has been done. Which value is being scaled against which value in the optimal solutions?

For the scaled differences in Figure 3, we first calculated the difference of the values for every iteration to the value of the optimal ordering and scaled the difference by the maximum difference.

L240-241: Here comes one example of my confusion with “exchanged metabolites”. In Fig. 3, is the number of exchanged metabolites referring to the number of metabolites secreted by each gap-filled model, or is it the total number of metabolites added to the minimal medium?

Here, the exchanged metabolites are meant as the total number of metabolites that are secreted by each of the community members. We updated the caption of Figure 3 and the text.

L252-253: “suggesting a dependence between these two criteria”. I am not sure I understand this correctly; the results suggest a dependence of the sum of biomass reaction fluxes and the number of exchanged metabolites added to the medium? I would have expected that to be the case, unless maybe if all models were already growing at their maximum capacity.

L260-262: What were the scores of the two reference models? Scores are only (partially) listed for B. metaterium in the text. For an audience not familiar with memote, what are good scores?

The problem with reporting Memote scores is that, unfortunately, often errors occur when models are analysed using this tool. We were not able to obtain a score for the *M. extorquens* AM1 model. We experienced that the total score reported by memote alone is not always informative, because metabolites, reaction, and gene annotation (i.e. cross-references) also influence the score. Hence, we also looked at the consistency scores and the fraction of reactions that can carry unlimited flux, which is part of the consistency score. Generally, we would consider scores above 60 to be good for a curated model. Concordantly, a consistency score of 60 or 70 would be good for a curated model. However, often scores drop to zero because some model features are not properly recognized (likely due to formatting issues, which are not easy to resolve). We saw this when investigating the reconstructions from the individual approaches. Since these issues are related to memote, rather than the usability of the models, we opted not to further expand on this part in the manuscript.

L277: Fig. 4B, the line types indicate the average similarity based on what? Reactions?

Here, we calculated the Jaccard Index between sets of added reactions.

L525-527: There's a maximum biomass flux, is there a minimum? Maybe the other end of the range; 2.8 1/h being very fast-growing bacteria, perhaps the other end could be constrained to very slow growing bacteria?

We thank the reviewer for this suggestion. Currently, we enforce a minimum biomass flux of $10^{-4}\ h^{-1}$. Evidence suggests that about 4% of bacteria in the wild have doubling times above 100 h, which relates to a growth rate of $6.9 \cdot 10^{-3}\ h^{-1}$ (10.1098/rspb.2018.0789, 10.1099/00207713-48-4-1383, 10.1016/0929-1393(94)90005-1). For the updated version of the manuscript, we increased the lower limit for growth to 10^{-3}.

Reviewer #2

Reviewer #2: In this work, Wendering and Nikoloski develop a community gap-filling algorithm that aims to identify a minimal solution set, while exploring 100 random gap-filling orderings, according to the following criteria: number of added reactions, dependence of the first member on exported metabolites of subsequent models, number of exchanged metabolites, and sum of biomass fluxes of all community members. The authors observed that the solution set obtained by COMMIT was significantly smaller than that obtained by gap-filling each model individually. This is a contribution that adds to a large body of literature that addresses similar challenges for individual models and microbial community ones. There is a number of key issues that remain unclear.

Major comments:

1) It is not surprising that the number of added reactions is lower as it is mathematically provable that allowing for cross-feeding between models will lead to reduction of the need of added reactions for connectivity. What is needed is to quantify whether the reduction reaches a particular threshold that would not have happened by chance. Possibly an F-test would be a solution.

We would like to thank the reviewer for this comment and suggestion. Although we value the idea, in our opinion, an F-test cannot be applied as we do not have a random case that would give us a distribution to compare our results with. What we did instead was to test for a difference in means between the individual and conditional gap fillings and found that their means are significantly different. Moreover, both individual and conditional approaches result in solutions that contain up to 37% reaction that are supported by sequence similarity.
One way to test whether the reduction could have happened by chance could be to add random reactions of the same sizes as added by the conditional gap filling approach and assess whether these render the models feasible. However, this will yield a highly significant result since most likely none of the random set can lead to a feasible model because the combinatorial explosion that results from choosing these sets from a large reaction database.
Prompted by the second point of the reviewer we assessed the performance of COMMIT with a well-described two-species community with published models (see answer to point 3, below).

2) The optimization formulation minimizes sum of molar fluxes. This de-emphasizes flows involving metabolites with large molecular weights possibly causing systematic discrepancies.

We understand that reviewer is concerned about large metabolites having an equal probability of being exchanged as small metabolites. This is, however, not the case since the classification for permeability includes a threshold for the molecular weight (i.e. 500 g/mol), which leads to a lower probability for the addition of exchange reactions with metabolites exceeding this value.

3) Gap filling algorithms including COMMIT are not unsupervised algorithms for fixing gaps. Each gap resolution strategy in the model needs to be scrutinized for biochemical evidence. In our experience,

gap-filling without any follow up leads to bloated models that propagate misannotated reactions in follow up investigations. The authors need to specify specific instances where the cross-feeding and/or strategy suggested by COMMIT was experimentally verified. Also, they need to caution for cases where COMMIT tends to make systematically erroneous suggestions. Absent of this, this effort is simply a computational exercise devoid of any potential for new insight.

We thank the reviewer for this critical comment. We are aware that gap-filling can lead to biologically improbable solutions. However, the premise under which our study was conducted was to create reconstructions with little available information. Therefore, we attempted to provide the most information base for the algorithm. Many of the added reactions for the two soil communities have genomic support (up to 37%). Thus, for this study, we see the gap-filling step as a way to build functional models while keeping the added reactions as few and accurate as possible, since we have no way to validate them for uncharacterized soil bacteria.
To test the performance of COMMIT with and without respecting the community composition, we employed COMMIT to fill gaps in models of a well-studied two-species community of *Desulfovibrio vulgaris* and *Methanococcus maripaludis* (10.1038/msb4100131). We utilized models published by Flowers et al. 2018 (10.1111/1758-2229.12619) and Richards et al. 2016 (10.1128/JB.00571-16). To this end, we removed reactions randomly from both networks. Here, we only allowed for a removal of flux-carrying, internal reactions, which could further be translated to MNXref namespace (341 for *D. vulgaris* and 304 for *M. maripaludis*). For 50 iterations, we calculated the precision and recall for COMMIT either applied individually or conditionally after removal of 1%, 2%, 5%, or 10% of the allowed reactions from the network. As a result, we obtained the following average precision and recall values.

		Desulfovibrio vulgaris		***Methanococcus maripaludis***	
	**% allowed reactions removed**	Precision (%)	Recall (%)	Precision (%)	Recall (%)
**individual**	1%	1.87	24.00	24.02	46.51
	2%	3.50	25.00	28.32	40.35
	5%	7.30	22.75	25.73	39.56
	10%	12.43	23.46	27.02	38.04
**conditional**	1%	2.02	24.03	25.53	48.06
	2%	4.23	26.67	28.33	40.00
	5%	7.97	21.18	29.93	40.80
	10%	14.54	23.14	28.37	39.11

These values are lower than those obtained by Latendresse and Karp (10.1186/s12859-018-2050-4), who used a similar procedure (precisions >70% and >80% and recalls of about 60%). In comparison, the MIRAGE gap-filling algorithm (10.1186/gb-2012-13-11-r111) showed precision and recall values of 41.9% and 24.3%, respectively. We are aware that the performance of COMMIT in recovering removed reactions is lower compared to more sophisticated approaches (e.g. Meneco

(10.1371/journal.pcbi.1005276)); however, we consider the performance as good enough given the tremendous advantage in computation time. Nevertheless, one future step towards improving COMMIT will explore different gap-filling algorithms.
We further applied the COMMIT function to find permeable metabolites to both models and found several candidate molecules that can be exchanged at low cost, i.e., N_2, acetate, carbamate, ethanol, formate, H_2S, nicotinate, succinate, and sulfate for *D. vulgaris*, while for *M. maripaludis*, we found 4-amino-2-methyl-5-(phosphooxymethyl)pyrimidine, 5-(2-hydroxyethyl)-4-methylthiazole, CO, methane, and selenite.

4) Gap filling almost always leads to the formation of thermodynamically infeasible cycles especially for community models. It is unclear whether there is a safeguard against this. Are there any unbounded fluxes?

The reviewer mentions an important downside that we have not addressed in COMMIT. We observe a high number in stoichiometrically-balanced cycles in the final models (Table S4). We are aware of gap-filling algorithm that consider this problem (10.1016/j.isci.2019.100783). The need for multiple steps in this approach, formulated as MILP, indicates the complexity of detection and removal of SBCs. Choosing such an algorithm a modification thereof, would tremendously increase the running time of COMMIT, making it unpractical for large communities. However, one can run subsequent analyses with additional constraint to avoid this problem. We repeated the analysis of growth reduction upon blocking each of the secreted metabolites with loop-law constraint and one-norm minimization using the COBRA toolbox function *optimizeCbModel* to avoid infeasible cycles in the solution. We saw that growth was still possible and was therefore not dependent on thermodynamically infeasible cycles. Together with additional changes to the approach, we found fewer exchanged metabolites and metabolic dependences.

5) The authors need to make the case why we need yet another algorithm for gap filling. What is new in the approach that is beyond a simple modification of existing approaches.

We would like to thank the reviewer for raising this point. In fact, the core of the COMMIT procedure is identical with the FastGapFilling algorithm as described by Latendresse et al. 2014. We used additional weights for e.g., exchange reactions for permeable metabolites. The novelty of COMMIT comes with the consideration of permeable metabolites between community members. Hence, COMMIT performs gap-filling based on a dynamically changing medium, while most other gap-filling approaches use a single, static medium (except for Medusa, 10.1371/journal.pcbi.1007847). This is a feature that has not been considered before but is highly relevant in microbial communities as the available nutrients depend on their composition. Despite the obvious fact that a higher number of available nutrients decreases the size of the gap-filling solution, this is precisely what is expected if nutrients are actively, or passively exchanged between community members.

6) L76-78: potentially misleading, as SteadyCom solves a set of linear problems iteratively, where the number of iterations is independent of the number of organisms in the community

We understand the point made by the reviewer and we updated the sentence in the revised version of the manuscript. However, SteadyCom does solve whole community optimizations iteratively until the accuracy for the community growth rate reaches a threshold. The number of iterations is independent of the number of species but the LP itself can easily get very large.

7) L423: since CarveMe is stated to generate a GSM model using a supplied medium, not clear how using a static medium is justified in this work as nutrients available to an organism will be a function of metabolites excreted by others in the community and the plant root exudates as well

With this sentence we wanted to emphasize that the reconstructions generated with CarveMe are functional with the associated medium, but not necessarily with another medium. For subsequent analyses, we removed exchange reactions from all reconstructions. The minimal medium was later added for gap-filling where it was enriched by secreted metabolites of the community members. We updated the sentence in the revised manuscript.

8) L439-443: why were different genome annotation tools (such as RAST and DFAST) used for different GSM reconstruction tools? How do the results differ if a single tool is used?

Metabolic reconstructions from different approaches show substantial differences in metabolite, reaction, and gene sets, as previously observed by Mendoza et al. 2019 (10.1186/s13059-019-1769-1). Since we investigate a large number poorly-studied organisms, we cannot rely on experimental data and manual curation to increase the quality of draft reconstructions. However, we made the same observation as did Mendoza et al., which led to the idea of combining draft reconstructions from multiple approaches to make use of complementary information content. One of the main reasons for differences in the draft reconstructions is the use of functional annotation methods, which are linked to biochemical reaction databases. RAVEN 2.0, KBase/ModelSEED, and CarveMe all use their own method for this. As the Pathway Tools software required a functionally annotated genome as a starting point, we chose DFAST as a fast and accurate method. This also provided yet another approach that could potentially increase the final information content in the consensus model.

9) The authors state that the models generated using automated tools are of high confidence but provide no comparison between simulated growth and experimental data. Have those models been validated? Can the models display growth individually? Or the only growth simulations performed were as a community? Did the predicted results quantitatively match experimental observations?

The predicted growth rates cannot be validated as no experimental growth rates are available. However, all of them reach the upper limit without any additional constraints and hence exceed expected growth rates **(10.1098/rspb.2018.0789)**.

We checked whether the final models could grow on the minimal medium but this was not possible for any of the models on their own. This observation suggests that rich medium, as done in the original study by Bai et al. 2015, may be needed (but the composition of such medium is also not well-specified).. Hence, our results demonstrate that the reconstructed models are community-dependent and reflect the community composition, as intended by the algorithm.

We also would like to note that the resulting reconstructions can be used outside of the community context. This can be done by either only applying the first step of COMMIT that entails merging the reconstructions from the four approaches, followed by gap-filling in another context of choice, or by gap-filling the models with multiple community compositions first and then taking the consensus of added reactions as the complete model for further analysis.

10) The biomass equation acts as a sink in metabolic models, and thus a primary determinant of network fluxes and capabilities. Since GSM models of two community members is available, how do the results change if the biomass reactions from these models are used instead of the universal biomass reaction from CarveMe reconstructions?

We thank the reviewer for this suggestion, which goes along similar lines as another point raised by the third reviewer. The aim of implementing an approach as COMMIT was to rely on a fully automated pipeline that can generate functional models (in the context of a community) that can be used for downstream analysis, eventually including manual curation steps. The addition of a different biomass reaction to the isolate models that the reviewer mentions would impose further manual work and involve a second gap-filling step since not all compounds of these reactions are included in the automatically generated model. We have performed additional analysis that involve using gram-specific biomass formulations as included in CarveMe. However, this resulted in a similar problem, that the chemically balanced and reduced gap-filling databased did not contain all required compounds from these biomass compositions. Thus, the gap-filling database would have to be manually expanded to enable usage of these biomass reactions. These steps are postponed for future work aimed at refinement of the models.

11) The motivation behind determining highly-permeable metabolites and treating them differently during gap-filling is clear, but how would the authors account for possible confoundments that differentiate a permeable metabolite from being available for consumption by other members in a community, such as intracellular concentrations, and role as a metabolic substrate?

The reviewer addresses and important point that we approached by solving a linear program after gap-filling each individual model. This LP maximizes the flux through the exchange reactions for highly-permeable metabolites and metabolites that have plasma membrane transporters in the model, while keeping the biomass above a threshold (here 90% of the optimum). We assessed the importance of the threshold by running COMMIT with lower bounds of 80% and 100% of the optimal biomass. As a result, the number of added reactions was slightly lower when 100% of the optimal

biomass were guaranteed the in the described LP. Still, many metabolites can be secreted at low cost or without any cost. A reason for the latter observation is that including sink reactions for intracellular metabolites can also increase growth by allowing them to be secreted and thereby avoiding potentially energy intensive degradation and export of downstream products.

12) L379-381: If the ordering of the gap-filling is what assigns a community member to be a ‘provider’ (by providing specific compounds to the community that cannot be produced by other members), can the authors comment on the inferences made about that organism’s role in the microbial community being biased by the gap-filling process?

Prompted by the reviewer’s comment we revised the formulation of these lines as follows:

“In addition, we hypothesized that the order in which models are gap-filled corresponds to their roles as ‘helpers’ or ‘beneficiaries’. However, we did not observe a significant correlation between the number of permeable metabolites per model and the order in which reconstructions are considered for gap filling by COMMIT. Therefore, we concluded that the reconstructions that are gap-filled first, in the considered orderings of community members, provide specific compounds to the community that cannot be produced by other members. We did not observe a reduction of biomass flux upon blocking of metabolites unique to the first five models in the optimal gap-filling order. Nevertheless, the permeable metabolites from the first five models contained 11 of the 15 exchanged metabolites, which can be produced and secreted at the lowest cost with respect to the number of added reactions. Hence, the roles of isolates as ‘helpers’ may be manifested in the gap-filling order by their ability to provide specific metabolites to other members.”

to explicitly specify our hypothesis and support for it in the in silico experiments.

We integrated the suggestions made by all reviewers by updating the parameters of the approach. After analysing the results, we found that that there is still no correlation between the ordering and the number of permeable metabolites. Further, we did not find a reduction in growth rate for the remaining models, upon blocking the uptake of the metabolites unique to the first five gap-filled models in the optimal ordering. We hypothesized that the position of isolates in the optimal gap-filling order would correspond to their roles as ‘providers’ or ’helpers’, and ‘beneficiaries’. However, we could not confirm this by the two analyses described above, but we found that the permeable metabolites from the first five models contain 11 of the 15 metabolites that are also taken up by other community members (i.e. exchanged). Hence, we conclude that the correspondence of the gap-filling order with the roles of 'helpers’ and ‘beneficiaries’ is not manifested in the number of permeable metabolites but rather in the importance on the metabolites themselves. This cannot be assessed by a reduction in growth upon blocking uptake of metabolites unique the first five models because subsequent models can also export these metabolites (none of the exchanged metabolites is unique to the first five models). This relates to the chicken-and-egg problem as subsequent models will only be able to produce certain compounds if other nutrients are already provided. Still, the first five models are able to provide already 11 of the exchanged metabolites at the lowest cost in terms of added reactions.

Minor comments:

1) L54: Suggest replacing ‘analyse’ for ‘analyses’
2) L525: Suggest explaining in further detail what ‘satisfying results’ entail
3) L256: Suggest specifying whether the biomass flux upper bound pertains to the entire community or to each of the individual consensus models
4) L567: It would benefit the readers if the definition of sink reactions was provided somewhere in the text.

All these points are addressed in the revised version of the manuscript.

Reviewer #3

Reviewer #3: In this paper, the authors present a new gapfilling tool COMMIT for automated large-scale modeling of microbial communities. This approach allows for gapfilling of consensus models at the community level. The authors then apply the approach to analyze microbial communities associated with Arabidopsis. They construct models for over 400 arabidopsis rhizosphere isolates using multiple model reconstruction approaches. They merge these models into consensus models. The commit approach then gapfills individual models one by one, expanding the gapfilling media based on the capabilities of each previous model gapfilling. This is an interesting and valuable approach as it will scale better than the community-level gapfilling employed by tools like KBase. COMMIT could thus support generation of much larger and more complex community models.

Overall, there is clear merit in the proposed interative gapfilling approach, as it does offer a means of gapfilling larger communities. The consensus approach to model building is more questionable. There is some question as to whether a larger model is necessarily better, and incompatibility and conflicts between different modeling approaches may introduce more problems than it solves.

All this said, many questions arise from the studies, methods, and data presented in the paper:

1.) Note, KBase already has tools for constructing and gapfilling community models, which can be constrained to specific abundances. This could perhaps be mentioned in the introduction. Again, as mentioned above, this approach gapfills the entire community at the same time and thus won't scale to large numbers of species.

We thank the reviewer for this addition. The introduction has been updated in the revised manuscript.

2.) Merging and reconciling models, particularly models constructed from different biochemistry databases like KEGG, ModelSEED, BIGG and MetaCyc can be extremely challenging because of differences in the representation of biochemistry among these sources. Many metabolites don't have structures and thus aren't matched well in resources like MNXref. In other cases, reactions differ by the level of lumping performed, the number of monomers in a polymeric compound, or compartmentalization of metabolites in the reaction. Did the authors encounter these challenges? How was this overcome?

For the reconciliation of metabolites, we fully relied on the matching provided in MNXref. With respect to reactions, we did not consider lumping as it is difficult to detect those reactions. We only considered the chemically-balanced part of the MNXref database and further excluded reactions that contain "n" or numbers >20 as stoichiometric coefficients as polymer-building reactions can easily be the source of introduced stoichiometrically-balanced cycles. Additionally, we excluded generic compartments MNXD4 and MNXD5 as these are photosynthetic compartments. Compartments MNXD3 and MNXDX were also excluded as these cannot be unambiguously assigned to cytosol or extracellular.

3.) Note, in building KBase models, the authors could have retained the AuReMe gene calls and identifiers and just had RAST reannotate the existing genes. KBase permits upload of existing gene calls in GFF and genbank format. This would probably be a preferable workflow for what the authors wanted to do.

For the creation of KBase models, we intentionally uploaded the genome sequence without gene calls, to make use of the full KBase pipeline including RAST annotation. We were however not aware that an upload of GFF files is possible for model generation in KBase (models were generated in June 2019).

4.) Was a single universal biomass used? Why not vary the biomass based on species (e.g. gram positive, gram negative, archaea)? Would the method be sensitive to this? Can the decision to use a single universal biomass be justified?

We thank the author for this suggestion. Currently, we use the universal biomass as published by Xavier et al. 2017 (10.1016/j.ymben.2016.12.002). We considered using gram-stain specific biomass reactions and we have the information available. During the preparation of the updated manuscript version, we included the gram-stain specific biomass reactions that are used in CarveMe. However, these included metabolite species that are not contained in the gap-filling database, partly because they were excluded since they are synthesized in lumped polymerization reactions that we intentionally excluded. Hence, gram-specific biomass formulations could not be used without manually adding reactions to the database, which would exceed the scope of this study.
Certainly, the addition of gram-specific biomass reactions will affect the method as additional compounds must be produced. However, these would likely largely be the same across all the gram-positive or gram-negative species. Since the universal biomass reaction that we use was already generated using gram-positive and gram-negative bacteria, we expect it to be a good compromise between different compositions found among bacteria.

5.) Did charge (at pH 7) come into play on the permeability prediction? Highly charged compounds would generally not be permeable.

This is another interesting point mentioned by the reviewer. We did not include charge explicitly in the permeability prediction, but it enters the prediction of XlogP values obtained from PubChem. Further, the topological polar surface area (TPSA) entered the prediction of missing XlogP values and permeability prediction. Despite pH being a very interesting factor to look at here, it would be a tremendous effort to include it, since also the models themselves would have to be altered and the protonation of model metabolites would have to be adjusted followed by mass-balancing of reactions. To directly include charge in the prediction of permeability, one would also have to find an appropriate threshold value for the charge of a metabolite.

6.) With consensus modeling, there's a major concern of adding many false positive reactions. Do the authors have some way to testing for this (e.g. comparing with Biolog data)? The comparison to published models performed is good, but data performance would be helpful as well.

Unfortunately, we do not have enough information available, e.g. transcriptomic data, that could be used for a validation. However, both conditional and individual gap-filling runs showed between 30% and 37% sequence support for the added reactions for the consensus model without CarveMe reconstructions and the KBase reconstruction. The full consensus reconstructions had mostly only uptake reactions added to them, so the genomic support ranged between 2.4% and 12.2%.

7.) The reaction counts from Raven are generally surprisingly high (significantly larger than many published models). This doesn't necessarily imply improved quality (but it might be). Can the authors perhaps explain what kinds of reactions Raven is annotating that other methods are missing? Are the reactions active? What kind of metabolism is represented?

RAVEN 2.0 models are already a consensus of two models (KEGG and MetaCyc), this increases the number of reactions and likely improves the quality but can also leads to duplicated reactions, which amount to 41% (assessed by translation to MNXref identifiers). We performed an analysis of pathway distributions by mapping E.C. numbers in the draft and consensus reconstructions to KEGG pathways. As a result, the distribution of major metabolic subsystems is very similar across all model types. We saw that the most prominent subsystems were "Carbohydrate metabolism" and "Amino acid metabolism", followed by "Metabolism of cofactors and vitamins", "Metabolism of terpenoids and polyketides", "Lipid metabolism" and "Biosynthesis of other secondary metabolites". The order of the latter four subsystems differed slightly among the reconstruction types but the not observe a distinct distribution of RAVEN 2.0 reconstructions.

8.) How much did the annotations of the models agree? Did they generally assign the same genes to the same reactions? Merging conflicting annotations in consensus models could cause problems. How did the authors handle this case?

During the reaction matching step in consensus generation, we compared GPR rules of matched reactions by (1) the contained genes and (2) the logical operators connecting them in a Jaccard Index-like score. (identical reactions: 0.68 and almost identical reactions: 0.60). We did, however, not explicitly quantify mismatches, i.e., genes that were assigned to a reaction in one model but to a different reaction in another model.

9.) How did the authors relate 16s data to their isolate genomes for which they built models? By species taxonomy matches? By blasting 16s sequences against isolate genomes?

Here, we are grateful to Ruben Garrido-Oter for providing the refined 16S sequences for the individual isolates that were used in the study (Bai et al. 2014, 10.1038/nature16192).

10.) Were any metrics used to determine if 100 iterations of the COMMIT algorithm was sufficient? Were solutions significantly different based on the order in which the gapfilling was performed?

We did not determine the number of iterations by a statistical reasoning. Since the number of possible orderings of 20 models already is enormous, the number is rather a choice for time efficiency. We observed that the individual orderings are not substantially different but there is always a best ordering with respect to the four (or fewer) criteria we apply.
